# Organoids for Modeling (Colorectal) Cancer in a Dish

**DOI:** 10.3390/cancers14215416

**Published:** 2022-11-03

**Authors:** Florian Rathje, Stefan Klingler, Fritz Aberger

**Affiliations:** Department of Biosciences and Medical Biology, Cancer Cluster Salzburg, Paris-Lodron-University of Salzburg, 5020 Salzburg, Austria

**Keywords:** organoids, intratumoral heterogeneity, cancer stem cells, tumor immune microenvironment, predictive in vitro models, CRISPR/Cas9 gene editing, personalized precision medicine

## Abstract

**Simple Summary:**

Despite remarkable progress in the treatment of cancer patients, the medical need for drugs with better efficacy is still unmet and high. In addition to accurate prediction of drug efficacy for individual patients, pathophysiologically relevant preclinical model systems with increased predictive power are urgently needed to reduce the high rate of clinical trial failure in oncology. Organoids grown from patient material represent exceptionally valuable model systems to mimic and study human diseased tissues such as tumors. Here, we elaborate an overview of innovative and advanced organoid model systems and highlight the exciting opportunities of organoids for personalized precision medicine and the field of immuno-oncology drug development.

**Abstract:**

Functional studies of primary cancer have been limited to animal models for a long time making it difficult to study aspects specific to human cancer biology. The development of organoid technology enabled us to culture human healthy and tumor cells as three-dimensional self-organizing structures in vitro for a prolonged time. Organoid cultures conserve the heterogeneity of the originating epithelium regarding cell types and tumor clonality. Therefore, organoids are considered an invaluable tool to study and genetically dissect various aspects of human cancer biology. In this review, we describe the applications, advantages, and limitations of organoids as human cancer models with the main emphasis on colorectal cancer.

## 1. Introduction

Colorectal cancer (CRC) ranks third in incidence and is among the top five in mortality among all cancer entities worldwide [1]. While CRC is mostly a disease of the elderly population and the incidence among individuals over 65 years of age is stable or decreasing in most countries [2], the incidence in individuals under 50 years of age is rising globally [3]. The epidemiologic risk factors for this so-called early-onset CRC are generally the same as for overall CRC including red and processed meat consumption, alcohol intake, obesity, inflammatory bowel disease, and genetic predisposition [4]. The timing of the exposure to such risk factors seems to be critical, as it is assumed that exposure during early life leads to a delayed effect on early-onset CRC incidence. The molecular and clinical features of early-onset CRC are as heterogeneous as of late onset CRC but also differential to the latter Together with the fact that the mortality of CRC is still very high, this calls for more research efforts to better understand the specific molecular characteristics and causes of early-onset CRC on the one hand, and to find more effective treatment options on the other hand. Improved predictive model systems closely reflecting the genetic and cellular complexity of human cancer biology are urgently needed to advance current treatment strategies, particularly with regard to immunotherapeutic approaches. Organoid technology opens up the possibility to cultivate primary cells donated by patients or healthy individuals in a three-dimensional manner for a prolonged time. This enables a thorough investigation and even manipulation of the organoids helping us to understand complex biological processes and to better evaluate novel therapeutics.

## 2. Organoids for Cancer Research

**Figure 1:**
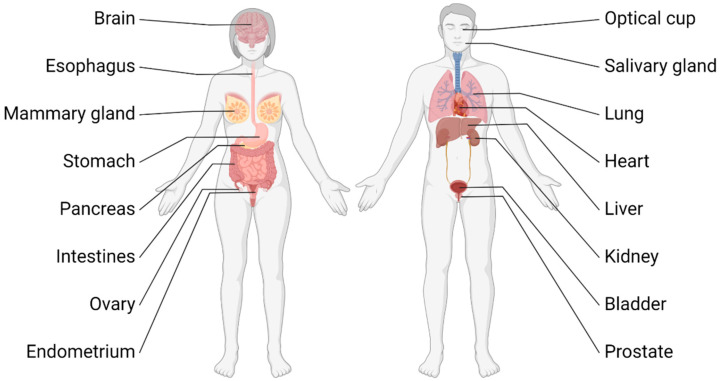
Overview of organs and tissues from which organoid cultures were generated.

For many years, research on different aspects of the development and disease of intestinal epithelium was dependent on immortalized cell lines or genetically engineered mouse models (GEMMs). The use of cell lines for translatable research is limited due to the genetic alterations these cell lines undergo during establishment and the missing three-dimensional context and cross-talks cells normally experience in the tissue. GEMMs, on the other hand, pose disadvantages as working with them is not only cost and time intensive but also raises ethical problems. The use of primary cells as an alternative eliminates several of these downsides. However, the cultivation of primary cells was for a long time limited to a few passages before the cells entered a non-proliferative senescent state. In 1987 some of these problems were circumvented by cultivating primary mammary epithelial cells from mice in a hydrogel called Matrigel enabling the cells to self-organize into three-dimensional ducts with lumen and to acquire physiological functions [5]. Matrigel is an extract from Engelbreth-Holm-Swarm (EHS) tumors in mice, whose matrix resembles the basement membrane and forms a clear hydrogel at 37 °C. The major components of this extract are comprised of laminin, type IV collagen, heparin sulfate proteoglycan, and nidogens [6]. In 2009, Sato and colleagues described in a landmark study the development of mouse small intestinal organoids when they cultivated either intestinal crypts, which contain the niche for Leucine-rich repeat-containing G-protein coupled receptor 5 positive (LGR5+) stem cells, or single-sorted LGR5+ cells alone in Matrigel [7]. They observed that the stem cells in the organoids give rise to transit-amplifying cells which in turn differentiate in all major cell types of the intestine. The epithelial cells of the organoids form a three-dimensional structure with a single cell layer displaying apical–basal polarity with the apical side facing inwards towards the lumen, thereby recapitulating the physiologic structure of the epithelium in the intestine. Two years later in a follow-up study, they demonstrated the ability to grow organoids long-term from mouse colon as well as from human small intestine and colon [8] and described the essential growth factors needed for organoid cultivation. These factors include Wnt-3A and R-Spondin 1, which are activators of the WNT-signaling pathway required for stem cell maintenance, epidermal growth factor (EGF) for stimulating proliferation, and Noggin to inhibit the BMP-pathway in order to prevent differentiation. Furthermore, they showed that organoids derived from mouse adenomas or human colorectal tumors were able to grow independently from some of these factors due to oncogenic mutations in the respective pathways. Generally, organoids can be derived either from adult stem cells (ASCs), which is the case when organoids are generated from intestinal crypts, from embryonic stem cells (ESCs), or from induced pluripotent stem (iPS) cells [9,10,11,12]. Unlike ASC-derived organoids, organoids from ESCs and iPS cells require certain developmental and lineage-specific signaling cues to establish specific lineages and/or organoid types. In addition to mammary [13] and intestinal organoids, many other types of organoids cultivated from a variety of tissues and organs were described in the past years: organoids were established for stomach [14,15], liver [16,17], pancreas [18,19,20], kidney [21,22], prostate [23,24], ovaries [25,26], endometrium [27], esophagus [8], bladder [28], lung [29], salivary gland [30], optical cup [31,32], and even brain [33] and heart [34] (Figure 1).

### 2.1. Organoid Technology in Comparison with Conventional Models

For a long time, tumor-derived cell lines, GEMMs, and patient-derived xenografts were the golden standard of cancer research and disease modeling. While all these models have their benefits and drawbacks, the emergence of organoids opened new and exciting possibilities. In this chapter, we discuss the advantages and disadvantages of (CRC-)organoids compared with existing tumor models. Two-dimensional immortalized cell lines are generally considered non-physiological, due to the immortalization process and lack of tissue architecture and complexity. Furthermore, most cell lines were established decades ago (e.g., HeLa cells in 1951) and consequently no longer faithfully represent their tissue or tumor of origin. Moreover, cell lines were propagated in many different laboratories under varying cell culture conditions around the world. Therefore, different batches of the same cell lines are not comparable with each other due to genetic drift [35], which may contribute to the reproducibility crisis in biological sciences [36]. GEMMs, especially conditional GEMMs, where an inducible Cre-recombinase is expressed under the control of a tissue-specific promotor, allow for spatiotemporal control of a gene knockout or induction of gene expression, and therefore enable functional in vivo studies [37]. Although GEMMs have contributed significantly to the validation of cancer genes, drug targets, and the characterization of treatment-resistance mechanisms, they also come with drawbacks. The generation of GEMMs is a time- and cost-consuming task and GEMMs do not fully recapitulate human conditions and physiology due to differences in mouse and human biology (e.g., genetics, immune system, microbiome). Thus, preclinical studies using GEMMs often failed to translate the findings to clinical settings raising questions about the predictive power of GEMMs. Xenotransplantation of patient-derived tumor material into immunodeficient mice (patient-derived xenografts; PDX) opens the possibility of interrogating human cancer cells in a complex microenvironment, thereby more accurately mimicking human cancer and enhancing the translatability of research. However, generation of PDX is often hampered by low engraftment efficiency as well as being labor and cost intensive. Furthermore, the necessity to use mice lacking an intact immune system prevents studies investigating cancer immunity including the assessment of immunotherapies. Moreover, studies interrogating the crosstalk of tumor cells and their microenvironment are affected by gradual replacement of human stromal cells by mouse equivalents as the tumors are serially passaged over time.

Organoids circumvent many of the limitations of the models mentioned above (Table 1). In contrast to cell lines, normal and tumor organoids can be derived from small samples of primary patient tissue materials such as needle biopsies, urine [22], and bronchial lavage, [29] often with high efficiency. Another advantage of organoids over cell lines is their feature to better recapitulate tissue architecture, (epi)genetic heterogeneity and cell types, and functions of their primary tissue. These aspects make them more relevant for disease modeling and drug response predictions. A possible drawback of organoids is their limited suitability for high-throughput screening of compounds or knock out libraries because the viscosity of extracellular matrix gels used for standard organoid culture conflicts with fast and automated sample handling. Therefore, some research groups investigated culture conditions without or with low percentage gel conditions to adapt organoid technology to high-throughput applications including microfluidic systems [13,38]. Another disadvantage of organoid technology and cultures compared with other models is the challenge of standardization. In particular, the dependence of organoid cultures on extracellular matrix hydrogels (i.e., Matrigel), which exhibit a high batch-to-batch variability in protein composition and density, poses a major problem in the field [39]. Moreover, the major laminin isoform of Matrigel is laminin-111 [40], which may not be optimal for culturing some types of tumors and/or their normal tissue counterparts. Hence, efforts are currently underway to design synthetic and defined PEG- or biopolymer-based matrices that support the growth of organoids, although they are still inferior to state-of-the-art hydrogels [41,42]. Moreover, the medium composition for standard organoid cultures is constituted in such a way that (cancerous) epithelial cells are positively selected over stromal cells. Therefore, in most tumor-derived organoids only the epithelial compartment of the cancer is present. There are ongoing efforts in the field to customize the medium composition allowing the reconstitution of pure epithelial organoid cultures with a variety of stromal and immune cell types. Overall, for organoids to become competitive and applicable for preclinical testing and studies, further enhancements regarding throughput, standardization, and costs are needed. However, once these limitations have been overcome, organoid technology will be indispensable as a bridge between low-complexity cell culture models and high-complexity in vivo models to advance personalized precision medicine.

### 2.2. Patient-Derived Biobanks of Living Organoids

Recent efforts have been undertaken in various cancer entities to generate biobanks of living organoids. The concept of an organoid biobank was examined first by Hans Clevers and colleagues. They used surgically resected normal and tumor tissues from CRC patients to establish patient-derived organoids (PDOs), which can be cryopreserved for an infinite time [43]. Many researchers have undergone efforts to create organoid biobanks from a wide range of cancers, including but not limited to CRC [43,44], breast cancer [13], glioblastoma [45], pancreas [46,47,48], prostate [49], bladder [28,50], esophageal adenocarcinoma [51], liver [52], and gastric tumors [53,54]. Establishment of PDOs is a successful and straightforward effort. Various studies reported over 70% success in creating PDOs [43,55,56]. This high success rate allows for the generation of stratified organoid biobanks representing the patient population with similar subtypes of various cancer entities compared with primary patient tumors [19,43,46]. Generally, PDOs are created from resected tumor tissue but recent studies highlight the feasibility of organoids established from fine needle biopsies. This modified protocol enables the generation of longitudinal organoid lines from the same patient, which reflect the changes in the tumors over time [28].

A great obstacle for establishing a living organoid biobank is contamination of the tumor organoids with healthy normal tissue since normal tissue outcompetes tumor tissue, most likely due to genetic instability and subsequent apoptosis of tumor cells [43]. However, normal tissue requires specific growth factors, which tumor organoids are independent of. Selecting for tumor organoids by removing those specific growth factors is a viable way of eliminating normal cell contaminations from tumor organoid culture [43]. Normal intestinal organoids require the addition of exogenous Wnt ligand for their growth, whereas over 90% of CRC patients carry mutations aberrantly activating the WNT-pathway [57]. Therefore, removal of exogenous Wnt allows for the specific selection of Wnt autonomous tumor organoids [43]. In Pancreatic Ductal Adenocarcinoma (PDAC) organoid culture, selection for specific tumor clones can be achieved by removing EGF for KRAS mutants, treatment with nutlin (MDM2 inhibitor) for TP53 mutants, or treatment with Bone Morphogenic Proteins (BMP) for selection of organoids with SMAD4 mutations [48].

Early studies in CRC were a proof of concept that an organoid biobank can be used for high throughput screens detecting tumor targeting drugs and discovering resistant subclones within PDOs [43]. Subsequent studies investigated the possibility to establish organoids in a reasonable amount of time in order to test standard of care therapy and stratify patients according to their response measured in the organoids. Treating CRC PDOs with either 5-fluorouracil (5-FU) as single agent or a combination of 5-FU, leucovorin and oxaliplatin (FOLFOX) yielded a range of responses, which predicted the therapy success in patients [56]. Another study, focusing on 5-FU, irinotecan, and irradiation, concluded with similar results, identifying vulnerabilities of PDOs towards single agent treatment; at the same time, the study uncovered resistant clones that were susceptible to combinatorial therapy [55].

### 2.3. Organoids as Tool to Study Intratumoral Heterogeneity and Cancer Stem Cells

Cancer is a highly complex and heterogeneous genetic and epigenetic disease, driven by mutations and epigenetic alterations in genes important for a wide range of cellular processes, such as regulation of cell proliferation, survival, metabolism, stemness, immune evasion, and DNA damage repair [58,59]. Though there are key genetic and epigenetic driver alterations characteristic of distinct cancer entities, there is striking heterogeneity between tumors in different patients and even within the same tumor, which is composed of multiple subclones with different (epi)genetic landscapes. These subclones develop and expand during tumor development and growth according to their fitness, which is determined by the gradual accumulation of driver mutations and epigenetic alterations [60,61]. The resulting mosaic of tumor clones with distinct malignant properties pose challenges for therapeutic interventions as rare and aggressive treatment-resistant clones can expand during and/or after therapy, thereby promoting the risk of relapse and disease progression [62,63]. It is thus of great importance to precisely elucidate and examine tumor subclones and single cancer cells, which is critical for predicting the response to targeted therapies and for reducing the risk of patient relapse. Consequently, predictive in vitro models of cancer need to maintain and reflect the heterogeneity of a patient´s tumor, ideally including the complex tumor-immune microenvironment [64,65,66,67,68,69].

The option to cultivate primary cancer cells from heterogeneous tumor tissues as tumor organoids makes this technology shine and superior to popular cell lines as well as transgenic mouse models, as organoids maintain the genetic and epigenetic intratumoral heterogeneity (ITH) in vitro. For instance, it was shown that gastrointestinal tumor organoids largely retain mutations of their tissue of origin [8,44]. To comprehensively recapitulate the clonal composition of a tumor, sampling of different tumor areas followed by the establishment of the representative organoid lines can be carried out. Notably, parallel cultures of organoid lines derived from different regions of the same tumor revealed up to 30-fold differences in drug response [70], underlining the importance of multiple samplings to capture ITH. Another comprehensive study investigating ITH was conducted by Roerink and colleagues in 2018 [71], where the authors sampled four to six different tumor sites from patients and established five to six clonal organoid lines for each tumor site. With this comprehensive set of clonal organoid lines and by comparing data to healthy wild-type organoids, the authors could build phylogenetic trees and show that clones from the same region shared common driver mutations but varied in overall mutation burden. They also observed inter-clonal differences in sensitivity to chemotherapeutic and targeted drugs.

**Figure 2:**
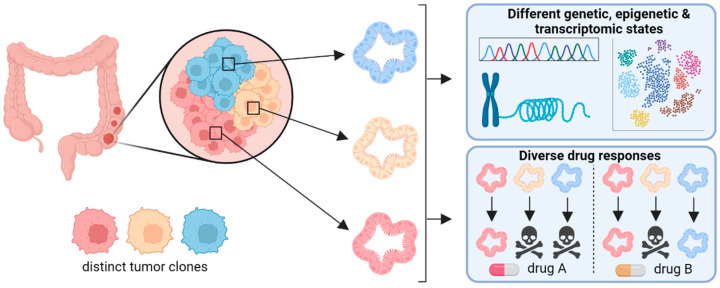
Organoids as tool to dissect intratumor heterogeneity. Generating organoids from various regions of an individual tumor enables to interrogate subclones for differences in the genome, epigenome, and transcriptome. The clonal organoids can also be tested for their sensitivity towards various drugs or drug combinations, therefore serving as surrogate system for personalized medicine.

Moreover, organoid cultures are not only suitable for cross-sectional studies, but also for longitudinal studies investigating the development of subclones over time. For instance, Ono et al. serially transplanted *APC*-deficient small intestinal organoids into recipient mice and analyzed the exome and transcriptome of bulk organoid samples and single organoid cells [72]. They showed that while the ITH regarding transcriptome increased after transplantations, the ITH of the exome decreased, indicating that signals from the tumor microenvironment can lead to the emergence of transcriptional subpopulations. The authors speculated that the reduced genetic heterogeneity could be due to microscale selection of tumor-initiating CSCs. In summary, these examples demonstrate that intestinal organoids are suitable for cross-sectional and longitudinal studies to dissect and understand ITH of CRC (Figure 2).

**Figure 3:**
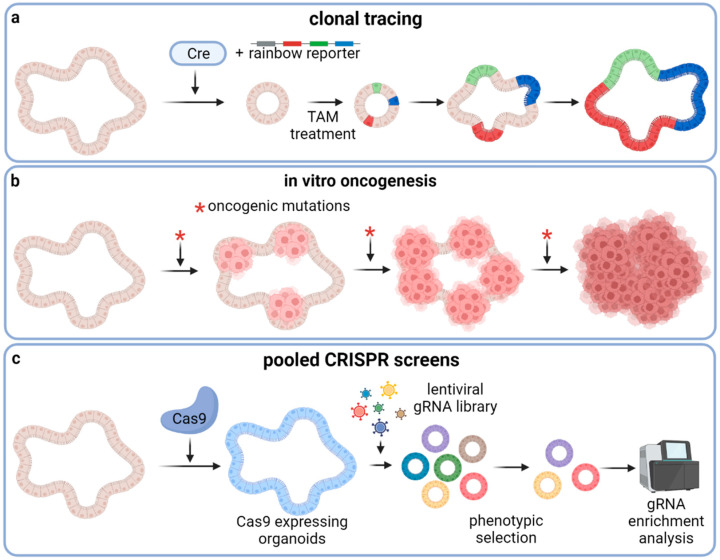
Gene editing applications in organoids. (**a**) Genetic knock-in of a rainbow reporter construct and a Cre-recombinase under the control of a stem-cell-specific promotor enables genetic tagging of individual stem cells and their descendants (**b**) Tumor development can be recapitulated in vitro by introducing oncogenic mutations to normal organoids, for instance by CRISPR/Cas9. Starting with non-malignant organoids, the step-wise addition of oncogenic alterations results in a panel of gradually more malignant organoids, enabling the study of tumorigenesis with its intermediate states. (**c**) Pooled genome-wide lentiviral CRISPR knockout screens in organoids are invaluable tools to identify candidate genes involved in complex biological processes driving carcinogenesis and/or resistance development. *: oncogenic mutations.

Organoids have also emerged as important tools to study the biology of rare cancer stem cells (CSCs) that have been shown to play a critical role in tumor initiation, growth, metastasis, and therapy resistance [73,74]. Like in vivo lineage tracing in genetically engineered mouse models, human CSCs can be studied by a comparable strategy in patient-derived organoids [75,76]. Shimokawa and colleagues traced LGR5+ colon stem cells in patient-derived colorectal tumor organoids xenografted to mice by knocking in a conditional Cre recombinase into the *LGR5* locus and labeling the organoids with a multi-color rainbow reporter (Figure 3a). Using this approach, the authors were able to confirm that, like murine Lgr5+ cells, human LGR5+ cells also act as CSCs with long-term self-renewal and differentiation capacity. However, ablation of LGR5+ cells only led to short-term tumor regression followed by tumor regrowth with re-occurrence of LGR5+ cells, indicating pronounced cellular plasticity with regard to replenishing the CSC population. Similar findings were made by de Sousa e Melo and colleagues by using a mouse-derived CRC organoid model [77]. Limiting dilution transplants of fluorescence-activated cell sorting (FACS)-isolated tumor cells revealed that tumor-initiating capacity was increased in the Lgr5+ cell fractions. Furthermore, Lgr5+ CSC depletion did not lead to tumor regression since Lgr5− cells were able to repopulate the Lgr5+ cell pool [77]. Recently, Ohta et al. identified LGR5^+^p27^+^ cells as dormant CSCs [78]. The authors generated fluorescent reporter PDO lines to visualize LGR5^+^p27^+^ in PDOs xenografted to mice. Chemotherapy treatment of these xenograft-bearing mice revealed that LGR5^+^p27^+^ CSCs are chemoresistant and exit dormancy after treatment in order to clonally expand. Most LGR5^+^p27^−^ cells went extinct during chemotherapy. Comparative transcriptome analyses of LGR5^+^p27^+^ and LGR5^+^p27^−^ cells revealed upregulation of pathways involved in cell adhesion. The authors identified COL17A1, a component of the hemidesmosome that adheres cells to the basement membrane and Matrigel, as a regulator of dormancy in LGR5^+^p27^+^ CSCs. They further showed that chemotherapy disrupts COL17A1 leading to activation of YAP signaling through focal adhesion kinase and subsequently to the exit of dormancy and regrowth of PDO xenografts. YAP inhibition delayed the regrowth of xenografts after chemotherapy. Contrary to this study, Cheung et al. showed that YAP activation in murine intestinal organoids suppresses Wnt-signaling, even in an Apc^−/−^ background, and leads to loss of stem cell properties [79]. Transplantation of engineered tumorigenic CRC organoids into mice and subsequent YAP activation led to loss of Lgr5^+^ cells and tumor regression. Furthermore, YAP activation reduced the growth of CRC PDOs. A further study demonstrating the capability of organoids to study cancer treatment persistence was conducted by Dhimolea et al. The authors used prostate and breast cancer PDOs and treated them with conventional chemotherapeutic agents [80]. PDOs were more resistant to these agents than cancer cell lines. Chemoresistant organoid cells adopt an embryonic diapause-like state and downregulate myc. Treating the PDOs with CDK9 inhibitors reverted this state and enhanced chemosensitivity. Organoid cultures are also suitable to investigate dedifferentiation and plasticity of epithelial cells. Hall et al. showed that the splicing factor Srsf1 is required for murine intestinal cell hyperproliferation and stem cell maintenance in an *Apc^fl/fl^*-background and for dedifferentiation of enterocytes in an *Apc^fl/fl^;Kras^G12D^*-background [81]. The authors demonstrated the effect on dedifferentiation by interrogating the capability of small intestinal villi, which should be free from stem cells, to form organoids. Villi isolated from *Apc^f/f^;Kras^G12D^;Srsf1^fl/+^* mice formed significantly fewer organoids than those isolated from *Apc^f/f^;Kras^G12D^;Srsf1^+/+^* mice. Taken together, these studies demonstrate that organoid models serve as valuable tools either to culture and genetically edit primary stem cells for downstream experiments or to use organoids directly as readouts for (cancer) stem cell properties.

### 2.4. Gene Editing of Organoids with CRISPR/Cas9

The advent of the CRISPR/Cas9 gene editing technology, which allows precise, fast, and cost-effective genetic manipulations of basically all cell types, enables scientists to mimic the genetic landscape of cancer cells and to perform large-scale genetic screens to rapidly identify, for instance, novel drug targets, resistance mechanisms, or oncogenic drivers [82]. In a seminal study using CRISPR/Cas9 in organoids, the Clevers group demonstrated that inactivation of the *APC*-locus by CRISPR/Cas9-mediated introduction of frameshift mutations in both murine and human intestinal organoids causes proliferation independently from the Wnt-agonist R-Spondin1, which is essential for the cultivation of wild-type intestinal organoids [83]. Furthermore, the authors were able to correct the disease-causing mutation in intestinal organoids derived from cystic fibrosis patients by repairing the cystic fibrosis transmembrane receptor (*CFTR*) locus with CRISPR/Cas9 and a mutation-corrected repair template. This seminal work was followed by many other studies involving CRISPR editing of organoids. Two independent studies expanded on the APC knockout in intestinal organoids and added other oncogenic mutations by CRISPR/Cas9 in a sequential order [84,85] to recapitulate colorectal tumorigenesis as proposed by the adenoma-to-carcinoma sequence model of Fearon and Vogelstein [86] (Figure 3b). In these studies, the authors knocked out the genes encoding APC, P53, and SMAD4 by introducing frameshift mutations and knocked in a constitutively active *KRAS*^G12D^ allele. By transplanting the genetically engineered organoids into recipient mice, the authors showed that these organoids grew out as human tumors confirming the tumorigenic effect of the introduced genetic alterations. In addition to conducting proof-of-concept experiments for gene therapies and in vitro tumorigenesis of wild-type organoids, CRISPR/Cas9 technology has been applied in intestinal organoids to demonstrate that KRAS mutation confers resistance to EGFR and MEK inhibitors [87] and in liver organoids to confirm the candidate tumor suppressor gene *BAP1* [88]. CRISPR/Cas9 was also used to study mutational signatures caused by loss of certain DNA repair genes. Drost et al. introduced loss-of-function mutations in the DNA mismatch repair (MMR) gene MutL homolog 1 (*MLH1*) or in the base excision repair gene Nth Like DNA Glycosylase 1 (*NTHL1*) in normal human colon organoids [89]. After two to three months of subsequent passaging, the organoids were subjected to whole genome sequencing. Organoids deficient in MLH1 accumulated base substitutions as well as insertions and deletions (INDELs) at a faster rate than unedited organoids and overall displayed a similar mutational signature as MMR-deficient CRCs. Knockout of NTHL1 revealed a different mutation signature than knockout of MLH1—one that closely resembles a signature found in a breast cancer cohort. Overall, this study showed that gene editing of DNA repair genes in organoids can be used to determine the mutational signature caused by other defects in the DNA repair machinery. Furthermore, gene editing has been applied in organoids for disease modeling beyond tumor models. Van Rijn and colleagues used CRISPR-engineered human duodenal organoids for genotype-phenotype association studies. They found that in duodenal organoids derived from patients suffering from congenital diarrheal disorders, the gene for diacylglycerol-acetyltransferase 1 (*DGAT1*) is commonly mutated and that these organoids harbor an aberrant lipid metabolism. CRISPR/Cas9-mediated knockout of DGAT1 in organoids from healthy donors led to comparable metabolic changes [90]. 

Pooled genome-wide lentiviral CRISPR/Cas9 knockout screens using a library of sgRNAs have become an extraordinarily useful tool to determine gene function within a defined biological context and setting (Figure 3c). While this method was widely applied in cell lines soon after its development, there are only a few publications with CRISPR knockout screen studies involving organoids due to more complex transduction protocols and higher cost factors regarding organoid culture. For instance, two studies used CRISPR screens to find genes that confer resistance to transforming growth factor β (TGF- β) induced cell death [91,92]. Ringel et al. found that only 4% of sgRNAs not targeting known TGF-β core pathway components conferred resistance when the screen was performed in human small intestinal organoids with an APC^WT^ background. However, by repeating the screen in APC-deficient organoids, the fraction of sgRNAs increased to 35%. Applying this approach with subsequent functional experiments, the authors were able to identify mutations in ARID1A and SMARCA4, two components of the chromatin remodeling SWI/SNF complex, conferring TGF-β resistance through altered chromatin accessibility resulting in attenuated TGF- β target gene expression. In another study, Michels and colleagues used a more focused sgRNA library in pre-malignant human intestinal organoids, which they transplanted into mice to expose the organoids to selection pressure from factors of the host microenvironment. In addition to the already known genes involved in the TGF-β pathway, they also identified several candidate genes so far not associated with CRC. 

In addition to intestinal organoids, organoids from other epithelial tissues have also been used for CRISPR knockout screens. Murakami et al. used the GeCKO sgRNA library on normal murine gastric organoids, conducted the selection in media with low Wnt concentration, and thereby identified novel modulators of Wnt-driven epithelial renewal such as Alk, Bclaf3, and Prkra [93]. Ungericht et al. used a Dox-inducible Cas9 to conduct a pooled genome-wide CRISPR-screen in iPS-derived kidney organoids [94]. Spatiotemporal control of Cas9 expression in combination with longitudinal sampling and endpoint sorting of tubular and stromal cells uncovered that rho-associated protein kinase (ROCK) inhibition improves mesoderm induction, confirmed that the genes *CCDC170* and *MYH7B* are involved in congenital anomalies of the kidney and urinary tract, and identified a list of candidate genes related to ciliopathies.

While genetic editing of organoids for disease modeling or elucidating the function of certain genes is already well established, pooled genome wide CRISPR knockout screens conducted in organoid systems will without doubt become an important and more frequently used discovery tool. The seminal studies already using such an approach have paved the way for future investigations to unravel the complexity of various biological processes, such as organ development and tumorigenesis, and to develop more effective drugs to combat malignant diseases.

### 2.5. Complex Patient-Derived Organoid Co-Cultures including the Tumor Immune Microenvironment

Although conventional organoid cultures represent an invaluable tool for studying the epithelial compartment of tumors, their pathophysiological relevance is limited with respect to the complexity of tumor-immune-microenvironment (TIME) interactions, which are decisive for malignant development and therapy [95]. Therefore, there is a very high need to establish organoid cultures also comprising cells from the TIME to better simulate the patients´ in vivo tumor setting and to improve the predictive power of organoid culture models. Tumor tissues present with varying numbers of tumor infiltrating lymphocytes (TILs) and myeloid cell types. New strategies of attempting to engage these immune cells in anti-cancer activity have been under intense research. The immune system plays a decisive role in the eradication of tumor cells, a process referred to as immune surveillance. During cancer progression, however, many of these surveillance mechanisms are inactivated or attenuated either by immunosuppressive signals mainly generated by malignant tumors or by cancer cell intrinsic measures resulting in immune evasion [96,97]. Harnessing the immune response against tumors, for instance, by immune checkpoint inhibitor treatments marked a breakthrough in modern oncology. However, low response rates and the difficulties of predicting responders is still a major challenge. Therefore, there is a strong need for model platforms that better recapitulate the tissue architecture of tumors including the TIME [98]. Directly culturing tumor fragments encompassing fibroblasts and pericytes, endothelial cells, and immune cells such as natural killer cells (NK), macrophages, dendritic cells, T-cells, and B-cells, allows for a fair representation of the TIME that is already pre-existing in the tumor [98]. These mixed PDO cultures preserve their inner composition of the TIME over several passages. However, within these cultures, TILs are lost over time and cannot be persevered for more than 60 days of culture [99]. Nevertheless, TILs originating from these mixed PDO cultures recapitulate the TCR repertoire and immune checkpoint functionality observed in primary tumor-derived TILs [99]. This demonstrates that mixed organoid cultures represent a valuable tool for immuno-oncology studies, despite demanding culture conditions and the culture duration. Alternatively, organoid cultures can be reconstituted with defined cell types such as autologous peripheral blood lymphocytes [100] or cancer-associated fibroblasts (CAFs) to study the complex signal interplay between cancer cells and individual components of cellular TIME [101,102,103].

TILs and autologous immune cells can destroy autologous organoids from the same patient, which makes organoids a promising and powerful future platform for testing the therapy efficacy of immune checkpoint inhibitors [100]. However, TILs can also react against components of the organoid culture, thereby attacking healthy controls in an unspecific manner by responding to an antigen present in the animal-based matrix organoids grow in [100]. To circumvent this problem, Dijkstra et al. omitted the ECM hydrogel during co-cultures. They demonstrated that whole colorectal cancer and non-small cell lung cancer organoids can be cultivated without ECM for up to three days in the presence of the anoikis inhibitor Y-27632.

CAFs have been identified as key players in the TIME by supporting the malignant properties and progression of cancer cells [104]. In organoid cultures, CAFs can enhance viability and proliferation of the individual organoids, while they do not necessarily increase organoid formation or initiation [102,103,105]. CAFs were shown to deposit components of the ECM, providing cues for cancer cell migration [106,107] and to secrete growth factors, cytokines, and chemokines known to promote cancer development [108]. In a liver tumor organoid model, inclusion of CAFs was shown to positively influence the size of organoids and their ability to engraft into an immune-compromised mouse model, where the CAFs closely surrounded the organoids [102]. The impact of CAFs on organoid viability is not limited to enhancing growth and engraftment. Several studies have shown that CAFs decreased the effectiveness of and increased the resistance to various treatment modalities, including standard chemotherapy and radiation [102,109,110]. Moreover, recent efforts in rectal cancer research identified a CAF subpopulation with an inflammation phenotype involved in conferring resistance to radiation [110,111]. CAFs, therefore, represent a crucial part of the TIME that needs to be included in advanced tumor organoid co-cultures.

In summary, co-cultivation of organoids with other cell types of the TIME enhances the biological complexity of these systems and subsequently requires more elaborate analyses to distill meaningful biological answers and findings. If all aspects regarding the set-up and evaluation of co-cultures are considered, such models will significantly improve our current understanding of the interplay between cancer cells and the surrounding components of the TIME. Such advanced models will be crucial for the establishment of predictive preclinical drug evaluation platforms urgently required to increase the likelihood of drug approval after clinical studies, which is dauntingly low in the field of oncology [112].

## 3. Conclusions

In recent years, organoid culture conditions were adopted to cultivate primary normal and cancer cells from several different organs, tissues, and tumors. Well-defined and molecularly characterized PDO biobanks for tumors and matched healthy tissues serve as invaluable resources for future studies in the field of drug development and personalized medicine. For many studies relating to human biology and disease, organoids are the in vitro model of choice, since they closely recapitulate physiological and cellular architecture, as well as biological and species contexts in an economic way. That said, there is still an urgent need to improve and overcome the limitations of organoid technology, including the lack of standardized ECM components by providing synthetic alternatives to the established animal-based hydrogels. Furthermore, solving—in the truest sense of the word—the high viscosity problem of Matrigel for high-throughput and microfluidics applications is an important step towards more economic organoid models that without doubt, will play a major role in the advancement of personalized precision medicine. The goal, however, must be to establish organoid-based models that capture and reflect the full extent of cellular heterogeneity, not only of the cancer cells, but also of the tumor-immune microenvironment. Progress and improvements in the predictive power of current pre-clinical test systems will to a large extent depend on their level of pathologically relevant complexity. In other words, the better the in vitro copy of the complex in vivo situation turns out, the higher the predictive power of the model systems in drug development and efficacy tests with primary patient samples will be. The further development of complex organoid models thus represents an important task for the benefit of many cancer patients.

## Figures and Tables

**Table 1:** Comparison of advantages and limitations of model systems in cancer research.

Model System	Advantages	Limitations
Cancer cell lines	Low cost	Non-physiological
	Ease of maintenance	Limited predictive power
	High-throughput capability	Variability between research groups
GEMMs	Suitable for functional in vivo studies	Generation is time and cost intensive
	Validation of cancer genes and	Not fully recapitulate human biology
	drug targets	Limited predictive power
PDXs	Good predictive power	Low engraftment efficiency
		Labor and cost intensive
	Human cancer cells in complex microenvironment	Limited suitability for immuno- oncology studies
PDOs	Easy to cultivate primary patient material	Limited suitability for high- throughput assays
	Recapitulates heterogeneity of tumor	Culture conditions difficult to standardize Challenging to establish cultures including microenvironmental components
	Easy to manipulate	
